# Rapid and label-free detection of gastrointestinal stromal tumor via a combination of two-photon microscopy and imaging analysis

**DOI:** 10.1186/s12885-023-10520-2

**Published:** 2023-01-10

**Authors:** Lianhuang Li, Xingxin Huang, Shichao Zhang, Zhenlin Zhan, Deyong Kang, Guoxian Guan, Shuoyu Xu, Yongjian Zhou, Jianxin Chen

**Affiliations:** 1 Key Laboratory of OptoElectronic Science and Technology for Medicine of Ministry of Education, Fujian Provincial Key Laboratory for Photonics Technology, Fujian Normal University, 350007 Fuzhou, P. R. China; 2 Department of Pathology, Fujian Medical University Union Hospital, 350001 Fuzhou, P. R. China; 3 Department of Colorectal Surgery, the First Affiliated Hospital of Fujian Medical University, 350001 Fuzhou, P. R. China; 4 Department of General Surgery, Nanfang Hospital, Southern Medical University, 510515 Guangzhou, P. R. China; 5 Department of Gastric Surgery, Fujian Medical University Union Hospital, 350001 Fuzhou, P. R. China

**Keywords:** Two-photon microscopy, Two-photon autofluorescence, Second-harmonic generation, Gastrointestinal stromal tumor, Tumor microenvironment

## Abstract

**Background:**

Gastrointestinal stromal tumor (GIST) is currently regarded as a potentially malignant tumor, and early diagnosis is the best way to improve its prognosis. Therefore, it will be meaningful to develop a new method for auxiliary diagnosis of this disease.

**Methods:**

Here we try out a new means to detect GIST by combining two-photon imaging with automatic image processing strategy.

**Results:**

Experimental results show that two-photon microscopy has the ability to label-freely identify the structural characteristics of GIST such as tumor cells, desmoplastic reaction, which are entirely different from those from gastric adenocarcinoma. Moreover, an image processing approach is used to extract eight collagen morphological features from tumor microenvironment and normal muscularis, and statistical analysis demonstrates that there are significant differences in three features—fiber area, density and cross-link density. The three morphological characteristics may be considered as optical imaging biomarkers to differentiate between normal and abnormal tissues.

**Conclusion:**

With continued improvement and refinement of this technology, we believe that two-photon microscopy will be an efficient surveillance tool for GIST and lead to better management of this disease.

## Introduction

Gastrointestinal stromal tumor (GIST) is one of most common mesenchymal tumors of human gastrointestinal tract. Over the years, it is reported that the incidence of GIST increases with the advancements in immunohistochemical staining techniques and improvements in diagnosis [1, 2]. As one of the most important factors widely accepted in predicting the biological behavior of GIST, tumor size is closely associated with prognosis in patients, for example, GIST with small size contributes to increase in survival [3]. Patients have favorable outcomes when the disease is detected at an early stage. Small‑sized GIST often has no any symptoms, and is not easy to be identified. At present, diagnosis of GIST mainly relies on morphological diagnosis and immunohistochemical assessment [4]. Although hematoxylin and eosin (H&E) and immunohistochemical staining are well-established techniques and have been demonstrated to be sufficient to diagnose GIST, these procedures are tedious, labor-intensive, and time-consuming.

Two-photon microscopy, a type of multiphoton excitation microscopy, is a nonlinear optical imaging technique and has matured into a powerful technology over the last few years [5–7]. This imaging technology allows label-free visualization of cells and extracellular structures with high resolution and little phototoxicity, and therefore helps basic and clinical researchers get detailed microstructural information of biological tissues. Previous findings demonstrated that two-photon imaging has great clinical potential for detecting disease and disease progression in cancer [8, 9], and has been widely accepted as a powerful tool to provide both qualitative and quantitative information in studying various diseases [10–12].

Here we try to introduce two-photon imaging—based on two-photon autofluorescence (TPAF) and second-harmonic generation (SHG) to detect GIST. We focus on imaging method that can be used to label-free identify different kind of tissue components in tumor microenvironment such as cells, collagen and elastic fibers, accompanying with an emphasis on automatic image processing strategy that can be used for quantitatively analyzing changes in collagen morphological features. Our study suggests that two-photon microscopy allows direct visualization of microstructural characteristics of tissues analogous to those obtained by histological method and therefore would be helpful to differentiate GIST from normal tissues. We hope this technology will become a rapid, reliable, auxiliary tool in the diagnosis of GIST.

## Materials and methods

### Patients and sample preparation

This study was approved by the Institutional Review Board of Fujian Medical University Union Hospital, and all patients signed an informed consent before the research. In this work, 30 fresh samples were collected including 15 gastrointestinal stromal tumors and 15 normal tissues. Moreover, 15 gastric adenocarcinomas were also collected for the sake of comparison. After removal by surgeons, every fresh sample without any processing (unfixed, unstained) was sent to pathology department immediately, and we cut two serial slices with 10 μm thickness by a cryostat microtome (Thermo Scientific CryoStar NX50, USA), where we used one section for two-photon imaging and stained another section with hematoxylin and eosin (H&E) to confirm experimental results. All the digital images of H&E-stained sections were obtained by a commercial whole slide scanner (VM1000, Motic, China).

### Two-photon imaging system

We used a commercial imaging instrument (LSM 880, Zeiss, Germany), which was combined with a mode-locked femtosecond Ti: sapphire laser (Chameleon Ultra, Coherent, USA), for obtaining two-photon images. In this work, a Plan-Apochromat 10× objective (NA = 0.45, Zeiss) was firstly used for whole-slide imaging and then a Plan-Apochromat 63× oil immersion objective (NA = 1.4, Zeiss) was selected to obtain high-resolution two-photon images of the regions of interest (ROIs). TPAF and SHG signals were detected in two-track channel mode under linearly polarized 810 nm excitation beam: one channel covered the wavelength range of 430–759 nm was used for collecting TPAF signal (color-coded with red) via a 32-channel GaAsP PMT array, while another channel covered the wavelength range of 394–416 nm was used for the collection of the backward SHG signal (color-coded with green) by a GaAsP photomultiplier tube (PMT). This imaging system takes ∼1.8 µs for collecting every pixel, and large-area images are obtained by a ZEN imaging software that could automatically assemble an array of two-photon images with 512*512 pixels.

### Image analysis

**Fig. 1:**
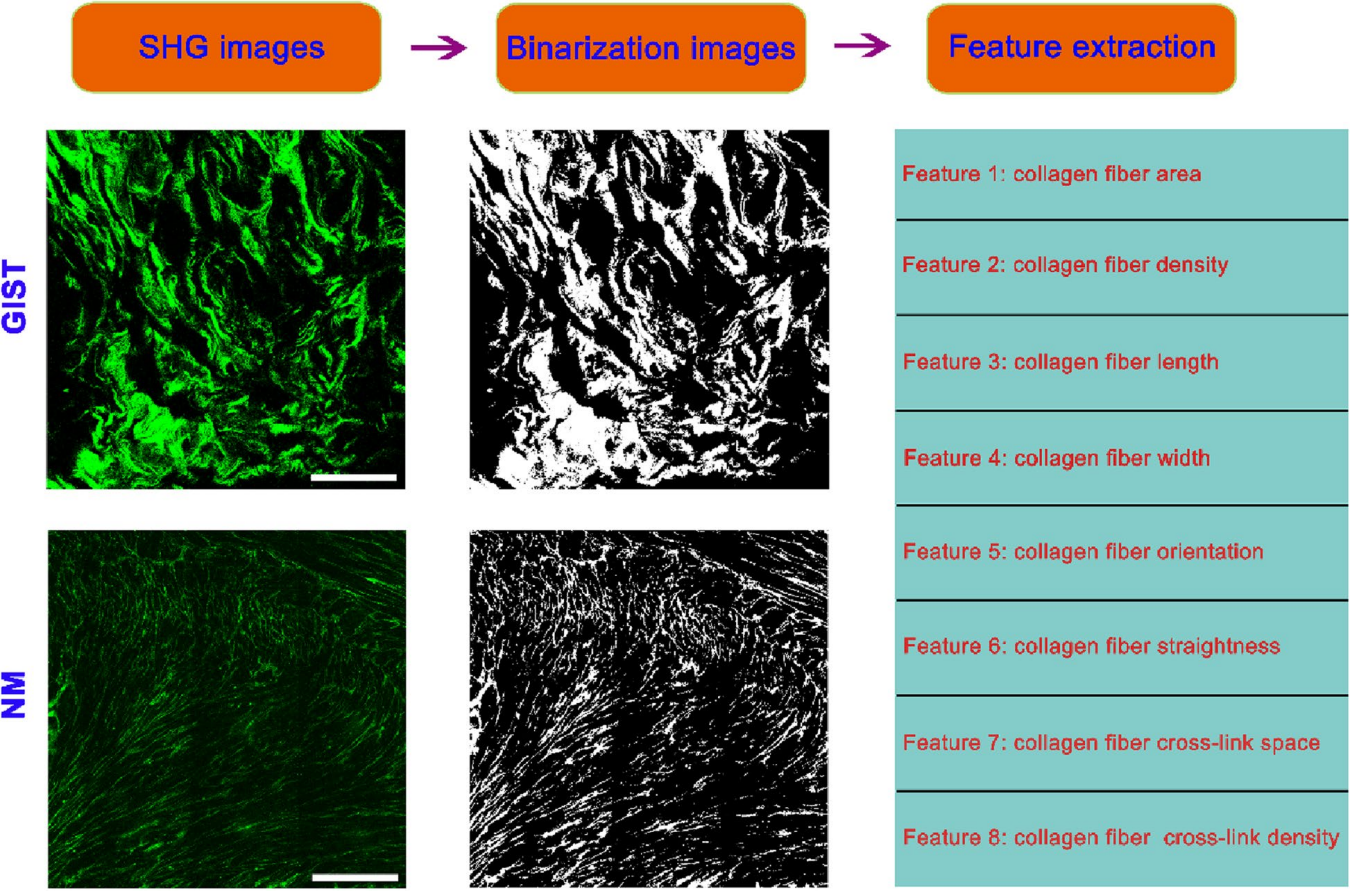
A schematic diagram of automatic imaging analysis to extract the morphological features of collagen fibers. Scale bar = 100 μm. NM: normal muscularis; GIST: gastrointestinal stromal tumor

An automated image analysis method which was performed using MATLAB 2016b was developed to extract the morphological features of collagen fibers from SHG images. As shown in Fig. 1, we selected the region of interest (ROI) of 1500*1500 pixels for quantitative analysis. For each sample, SHG images as the input images were first filtered by the Frangi filter to enhance the collagen fiber structures from noisy background, and then the enhanced images were segmented into collagen fibers and background by a segmentation algorithm based on Gaussian mixture models [13, 14]. The morphological closing and hole-filling were performed to smooth the binary mask of the collagen fibers, and any segment with less than 5 pixels was removed. In this work, a well-established fiber network extraction algorithm called “Fire” [15] was used to track and identify all potential collagen fibers reflected in SHG images, and then a series of ordered vertex sequences [16] were constructed to calibrate the skeletons of collagen fibers (any common vertices of different sequences were defined as cross-link points between collagen fibers). As a structured representation of collagen fiber networks, the ordered vertex sequences were used to quantify collagen morphological features including collagen fiber area (a.u.), density (fiber number per mm^2^), length (µm), width (µm), orientation (a.u.), straightness (a.u.), cross-link space (µm), cross-link density (a.u.) as previously described [13]. Quantitative results were presented using means with standard deviations (SD).

### Statistical analysis

All statistical analysis was processed using the IBM SPSS Statistics 21 program. The student’s t-test was chosen for evaluating the statistical significance, and *P-*value less than 0.05 was regarded to be statistically significant.

## Results

### Label-free identification of GIST by two-photon imaging

**Fig. 2:**
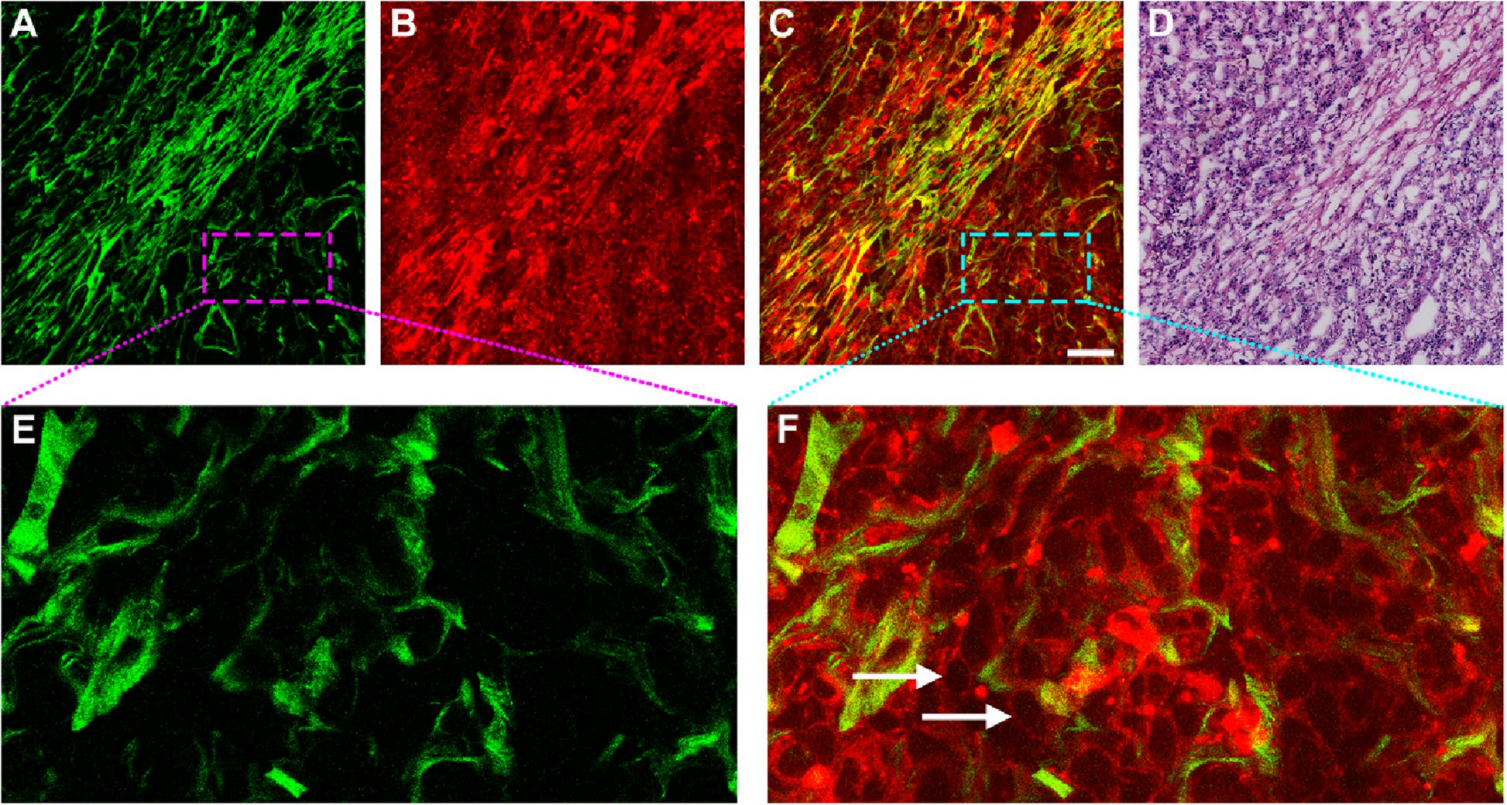
Two-photon images and the corresponding H&E-stained image of gastrointestinal stromal tumor. Scale bar = 100 μm. (**A**) SHG image (color-coded green), (**B**) TPAF image (color-coded red), (**C**) Composite image (SHG + TPAF), (**D**) H&E-stained image, (**E-F**) Zoomed images of the regions of interest (pink and cyan boxes, respectively). White arrows: GIST cells

GIST is the most common malignant mesenchymal neoplasm, and accurate diagnosis of this lesion is currently based on histopathologic examination of endoscopic biopsy specimens. However, this procedure is costly, labor-consuming and time-costing. Numerous studies have shown that two-photon imaging method is rapid, sensitive, reproducible, and especially SHG imaging offers a new way to guide region of interest selection for quantifying collagen fibers in different biological tissues [17, 18]. In this work, TPAF and SHG imaging were combined to image healthy gastric tissue, GIST and gastric adenocarcinoma ex vivo samples to perform a morphological characterization. As shown in Fig. 2, two-photon images clearly present the tissue architecture details of GIST. Specifically, SHG imaging (Fig. 2 A, E) allows users to visualize collagen distribution in tumor microenvironment, and TPAF imaging (Fig. 2B) shows that tumor cells appear with dark nuclei (white arrows in Fig. 2 F), and composite image (Fig. 2 C) could let users visually observe the spatial distribution of tissue components such as the cellular environment within the collagen matrix. All these features correspond to the H&E-stained image (Fig. 2D). Hence, this technique may provide new opportunities for revealing the relationship between tumor cell behavior and the role of collagen fibers in regulating such cell behaviors.

**Fig. 3:**
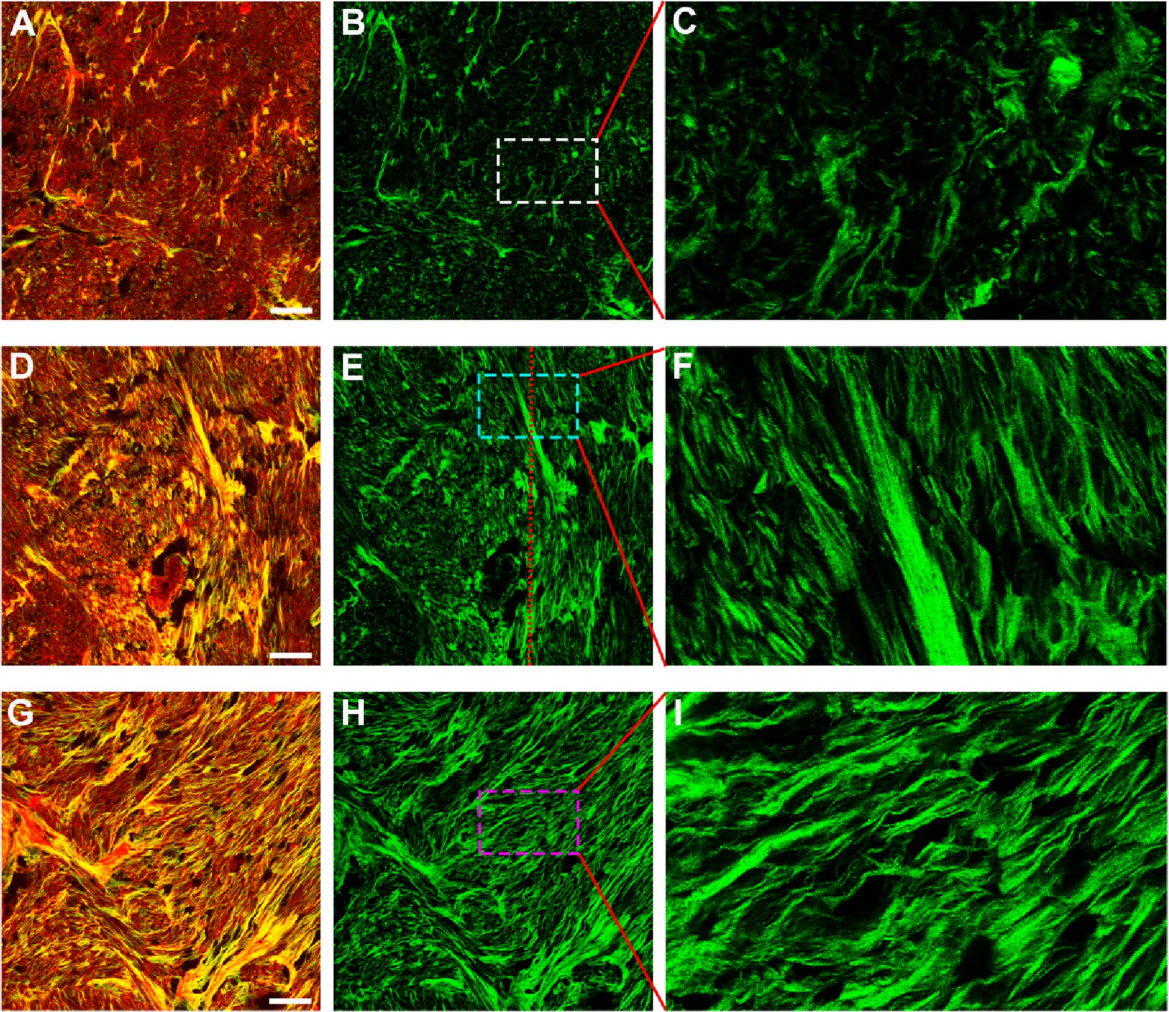
Two-photon images of different desmoplastic reactions caused by the invasion of gastrointestinal stromal tumor. Scale bar = 100 μm. (**A-B**) Composite and SHG images of mild reaction, (**D-E**) Composite and SHG images of moderate reaction, (**G-H**) Composite and SHG images of severe reaction, (**C, F, I**) Zoomed images of the regions of interest (white, cyan and pink boxes, respectively)

Generally speaking, tumor invasion will cause desmoplastic reaction, and some studies demonstrated that histological categorisation of the desmoplastic reaction is significantly associated with the prognosis of colorectal cancer patients with or without preoperative chemoradiotherapy [19, 20]. The presence of collagen fibers is therefore considered to influence the development of cancer. Our experimental results show that two-photon imaging has the ability to accurately and quickly monitor desmoplastic reaction induced by GIST, and even could directly discern different levels of response (Fig. 3 A, D, G). Specifically, for the type of mild reaction (Fig. 3B, C), collagen fibers are sparse, disordered, and fragmented in the tumor microenvironment; by contrast, there are abundant and directionally distributed collagen fibers for the severe desmoplastic reaction (Fig. 3 H, I). On the basis of these two reactions, we could recognize the moderate response (Fig. 3E, F) in which some collagen fibers are chaotic (left side of the red line in Fig. 3E) and some are orderly aligned (right side of the red line in Fig. 3E). Although it is not clear whether desmoplasia (tumor fibrosis) is a direct or an indirect indicator of GIST, the results of our research suggest that SHG imaging is of value in identifying different kinds of desmoplastic reaction.

### Comparison between GIST and gastric adenocarcinoma

**Fig. 4:**
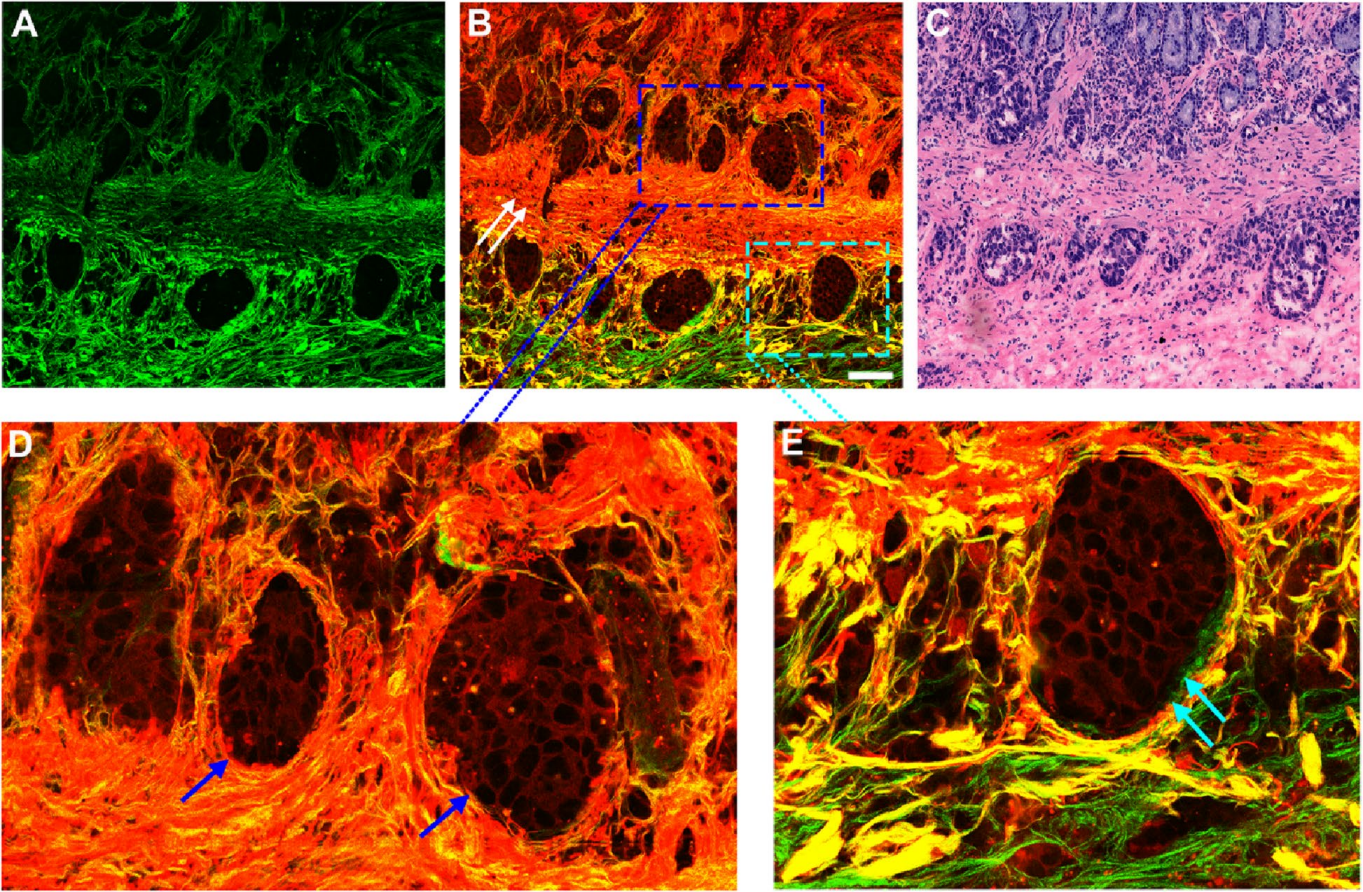
Two-photon images and the corresponding H&E-stained image of mucosa and submucosa invaded by gastric adenocarcinoma. Scale bar = 100 μm. (**A**) SHG image, (**B**) Composite image, (**C**) H&E-stained image; (**D-E**) Zoomed images of the regions of interest (blue and cyan boxes, respectively). White arrows: muscularis mucosae; blue arrows: gland-like tumors in mucosal layer; cyan arrows: adenocarcinoma cells with nest-like structure in submucosal layer

For the comparison and analysis, we further investigated the structural characteristics of different samples, finding differences between gastric adenocarcinoma and GIST. The histological structure of stomach is mucosa, submucosa, muscularis and serosa from the outside to the inside. Figure 4 presents two-photon images of gastric mucosa and submucosa with the invasion of adenocarcinoma. Imaging data reveals that GIST and gastric adenocarcinoma have completely different tissue architectural features. The muscularis mucosae (white arrows in Fig. 4B) separate the mucosa from submucosa, and in particular, adenocarcinoma cells surrounded by collagen fibers (Fig. 4 A) appear with nest-like architecture and tightly pack together (blue arrows in Fig. 4D). These tumor cells are totally different from GIST cells which are widely spread in the process of infiltration. Interestingly enough, it is found that adenocarcinoma cells with nest-like structure (cyan arrows in Fig. 4E) have infiltrated into the submucosal layer and were surrounded by collagen fibers. These structural characteristics are in excellent agreement with the digital image of H&E-stained adjacent tissue section (Fig. 4 C).

**Fig. 5:**
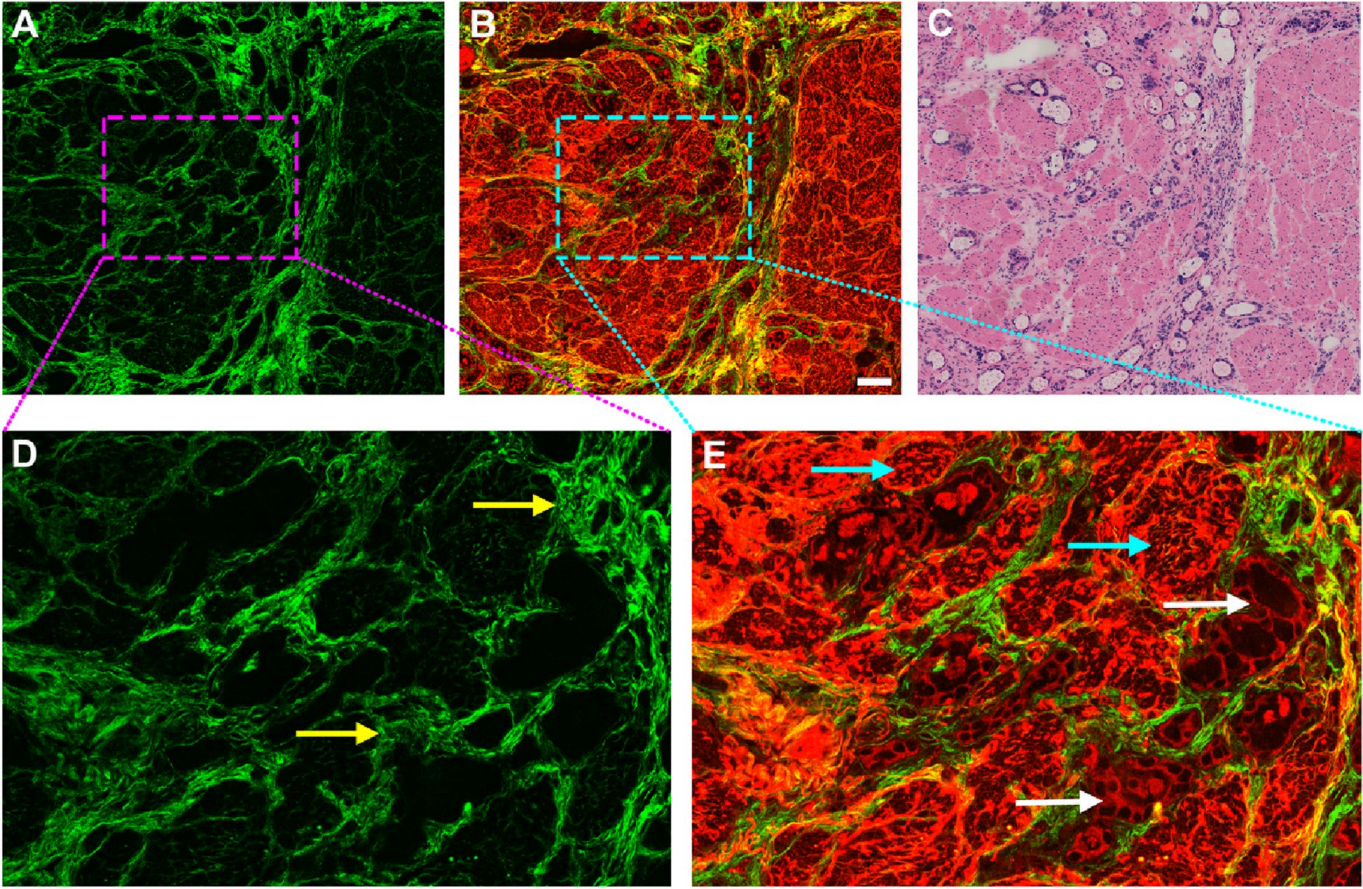
Two-photon images and the corresponding H&E-stained image of gastric muscularis invaded by adenocarcinoma. Scale bar = 100 μm. (**A**) SHG image, (**B**) Composite image, (**C**) H&E-stained image, (**D-E**) Zoomed images of the regions of interest (pink and cyan boxes, respectively). Yellow arrows: collagen fibers; white arrows: adenocarcinoma; cyan arrows: broken muscularis

In Fig. 5, it is clear that the collagen content within gastric muscularis propria is altered with the tumor invasion in the SHG images (Fig. 5 A, D). A lot of collagen fibers (yellow arrows in Fig. 5D) emerge because of desmoplastic response, and thus the detection of SHG signal from collagen fibers could provide us an approach to differentiate normal from abnormal tissues. Cancer progression is often associated with the destruction of normal tissues. TPAF imaging has the power to make us directly visualize the infiltrating adenocarcinomas and broken muscular tissues (cyan arrows in Fig. 5E). Two-photon images (Fig. 5B) demonstrate that normal muscularis has been damaged, becomes fragmented, and are gradually replaced by gland-like tumors (white arrows in Fig. 5E). These tissue architecture details readily correlate with the H&E-stained image (Fig. 5 C).

**Fig. 6:**
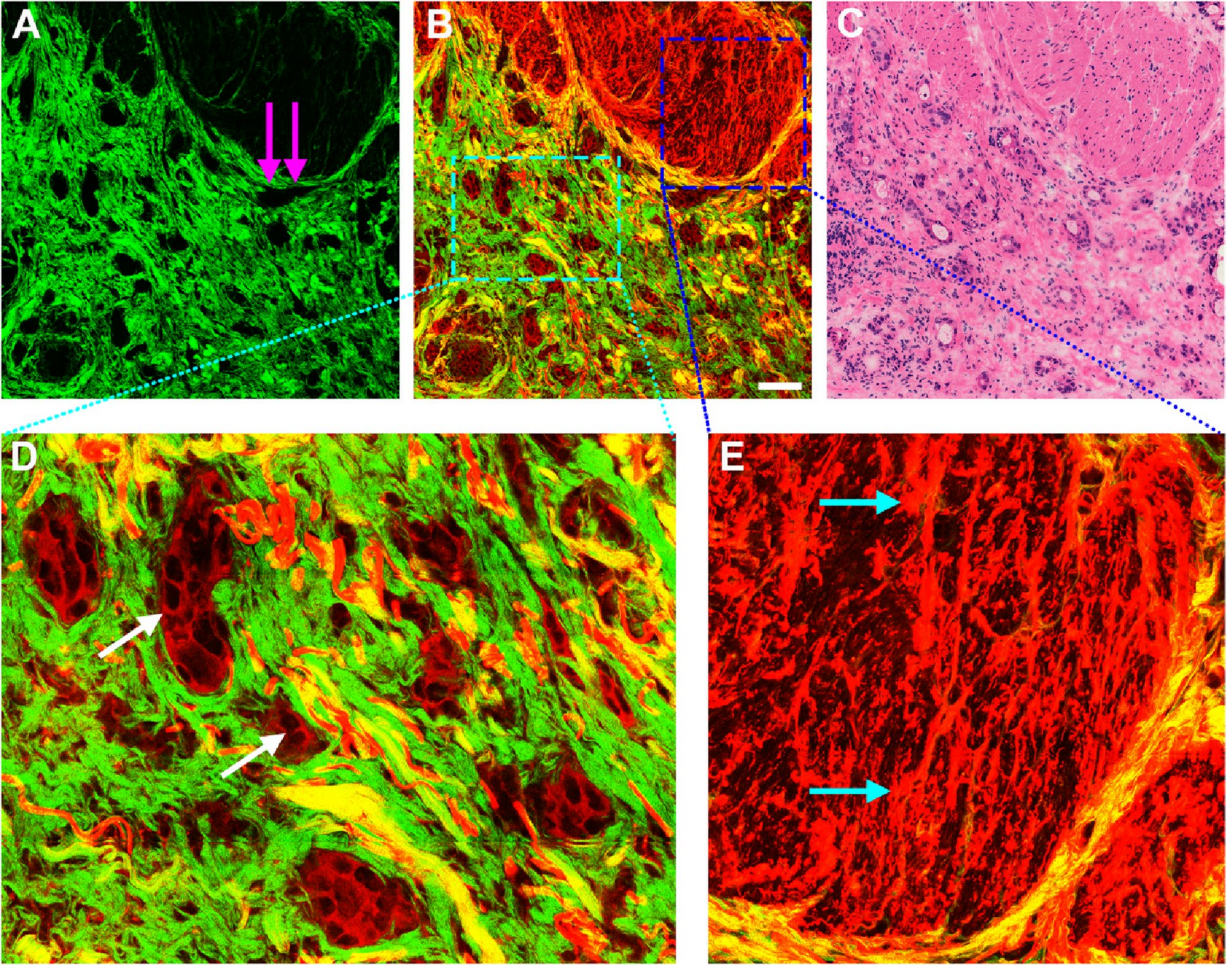
Two-photon images and the corresponding H&E-stained image of gastric serosa invaded by adenocarcinoma. Scale bar = 100 μm. (**A**) SHG image, (**B**) Composite image, (**C**) H&E-stained image, (**D-E**) Zoomed images of the regions of interest (cyan and blue boxes, respectively). Pink arrows: boundary between the muscular and serosal layer; white arrows: gland-like tumors; cyan arrows: elastic fibers

As displayed in Fig. 6 A, SHG image reveals that gastric serosa mainly composes of collagen fibers. However, there are many tumors with nest-like structure (white arrows in Fig. 6D) too in the serosal layer by the detection of TPAF signal, indicating serosa invasion in gastric cancer. The H&E-stained image of adjacent section (Fig. 6 C) is used for confirming these experimental results (Fig. 6B) obtained by two-photon microscopy. It is surprising that the proliferation of elastic fibers turns up in residual muscular tissues (Fig. 6E). The abnormal elastic fibers are fractured or gather together (cyan arrows in Fig. 6E), which are different from normal elastic fibers with a long rope-like morphology. Changes in tumor microenvironment may accompany disease progression, and maybe TPAF imaging will become an alternative tool for monitoring unusual conditions induced by tumor invasion without labeling. It seems that the tissue structures of GIST are entirely different from those of gastric adenocarcinoma based on the imaging results, and therefore could be identified by two-photon imaging.

### Quantitative analysis

**Fig. 7:**
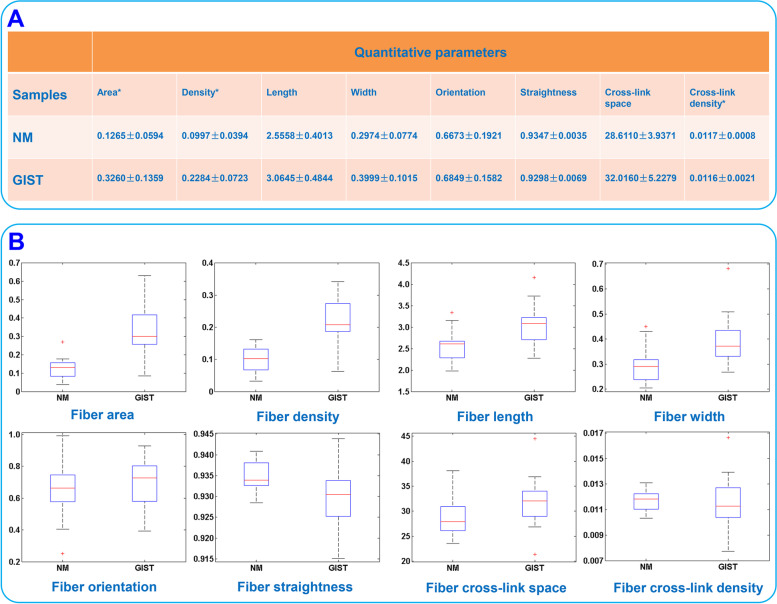
(**A**) Quantitative assessment of collagen features by automatic image analysis, (**B**) Data distribution of the eight morphological features of collagen fibers from NM and GIST, respectively. NM: normal muscularis, GIST: gastrointestinal stromal tumor. * denotes *P* < 0.05

Morphological characteristics alone are not sufficient to precisely identify GIST as they do not provide quantitative information about the changes within the extracellular matrix. It has been suggested that there is a relation between cancer risk and collagen alterations [17, 21]. Hence, in this work, we analyzed and extracted 8 morphological features of collagen fibers in the tumor microenvironment of GIST by automatic image processing strategy. GIST often originates from the muscle layer, and therefore we also extracted the 8 collagen characteristics from normal muscularis propria for comparison. The means with standard deviations and data distributions of these collagen features, including fiber area, density, length, width, orientation, straightness, cross-link space and cross-link density, are presented in Fig. 7 A and 7B respectively. Quantitative analysis shows obvious difference in collagen fiber area, density, and cross-link density between the normal muscularis (NM) and GIST. Collagen structure and organization in the microenvironment are potentially key determinants of tumor cell behavior, and the three parameters may be treated as indicators to distinguish healthy from diseased tissues. In summary, our method of two-photon imaging coupled with automated image analysis is sensitive, robust, and offers a new opportunity to quantify collagen fibers in different tissue samples.

## Discussion

Gastrointestinal stromal tumor (GIST) may arise anywhere in the gastrointestinal tract and is considered to be a potentially malignant tumor [22, 23]. GIST is a rare tumor, but the incidence has been increasing in the past few years. Previous research has shown that the metastatic risk of GIST increases with the increase of tumor size [24]. The prognosis of GIST is closely associated with early histological diagnosis. Currently, histopathologic examination on GIST is mainly dependent on the combination of H&E staining and immunohistochemistry, where H&E staining is used for identifying morphological features and immunohistochemical methods is used to visualize specific protein expression [25]. There are some problems in diagnostic process, such as heavy working intensity, need for time and experience. Thus, it will be meaningful to develop a new approach for assisted identification of GIST.

Two-photon microscopy is a powerful tool for imaging and exploring living cells, biological tissues as well as freely behaving animals at high resolution. For instance, some researchers have used this imaging technique for investigating morphological and metabolic changes in diseased corneas, hearts, breast and gastrointestinal tract [26–29], and Sun et al. utilized multiphoton imaging to study extracellular vesicles (EVs) in breast tumor microenvironment and found that EV density from breast cancer tissues was significantly higher than those from normal tissues [30], and recently, Zong et al. have developed a miniature two-photon microscope for brain imaging in freely behaving mice [31]. These previous studies suggest that two-photon microscopy may be utilized for the detection of GIST, and the assessment of GIST may be improved by monitoring tumor cells and the structural organization of stroma via two-photon imaging.

There are many intrinsic fluorophores in cells such as NADH, FAD, and thereby TPAF imaging is capable of directly detecting individual tumor cells in GIST (white arrows in Fig. 2 F) without any exogenous contrast agent, which are completely different from adenocarcinoma cells as these cells often appear with gland-like structure. Elastic deposition (Fig. 6E) induced by tumor invasion is found in the muscularis propria via TPEF signal, for example, the content of elastic fibers obviously increases, and these fibers are fractured, short and thick. Moreover, the four-layer structure with mucosa, submucosa, muscularis, and serosa is well presented by the combination of TPEF with SHG imaging. These imaging results will enable two-photon microscopy to monitor the development of diseases that is useful for individual decision-making with respect to treatment strategy.

As the role of collagen fibers in cell behavior and tissue homeostasis becomes more apparent, new techniques are required to detect subtle changes in collagen organization beyond gross content. Previous reports revealed that the type of desmoplastic response has close relation with patient prognosis, and histologic categorization of desmoplastic reaction is an independent prognostic factor in several kinds of tumors, for example, in colorectal cancer, oesophageal squamous cell carcinoma, cervical squamous cell carcinoma [19, 32, 33]. In this work, we show that SHG imaging could provide the necessary resolution to visualize detailed collagen changes in tumor microenvironment and directly recognize different kinds of desmoplastic reactions (mild, moderate, and severe) from fresh tissues. Additionally, paraffin samples after deparaffinization could be used for two-photon imaging too. Thus, two-photon microscopy may be a potentially useful tool for extending our understanding of GIST biology and predicting prognosis of patients.

We also introduce a new method that successfully combines SHG imaging with automatic image processing to target specific regions of interest for quantitative analysis and thereby could increase our ability to understand the interactions between tumor cells and their collagenous environment. We obtain 8 morphological features of collagen fibers including fiber area, density, length, width, orientation, straightness, cross-link space, and cross-link density from GIST and normal muscularis propria. Statistical analysis shows that there is significant difference in fiber area, density and cross-link density. Tumor invasion often causes collagen reorganization such as desmoplastic response, and therefore collagen fibers in tumor microenvironment would increase and become thick and more chaotic, which leads to the difference in the three characteristics of morphology, and increased collagen density would further promote tumor progression [34]. These three variables may be treated as optical biomarkers for distinguishing normal from abnormal tissues. Of course, there are some limitations of this study: firstly, we are unable to explore the influence of the polarization state of the excitation beam on the SHG imaging of tumor microenvironment and to carry out forward direction SHG imaging because of the limitation of our imaging system; secondly, we cannot collect enough samples to investigate the spatial heterogeneity of GIST.

## Conclusion

In summary, our results demonstrate that two-photon microscopy is indeed a powerful, informative tool for detecting GIST and monitoring microstructural changes in tumor microenvironment. At present, two-photon imaging technique is rapidly developing towards being fast, portable, miniature, and inexpensive. We foresee that this technology will play an important role in helping clinicians achieve an accurate diagnosis in the era of precision medicine.

## Data Availability

The code to extract the collagen features is available at [https://github.com/qldqq1984/CollagenFeature].
